# Intergenerational Day Centers: A New Wave in Adult and Child Day Care

**DOI:** 10.3390/ijerph20010809

**Published:** 2023-01-01

**Authors:** Neda Norouzi, Jacqueline L. Angel

**Affiliations:** 1 School of Architecture + Planning, The University of Texas at San Antonio, San Antonio, TX 78703, USA; 2 LBJ School of Public Affairs, The University of Texas at Austin, Austin, TX 78249, USA

**Keywords:** Intergenerational Day Centers, adult day care, child development, architecture, urban reality, intergenerational design

## Abstract

Intergenerational Day Centers (IDCs) are an innovation that addresses two important societal challenges, the continuing need for childcare and the emerging demand for older-adult supportive services that help them remain independent in their homes. These facilities provide care, and specialized resources and activities for both older adults and children in one location. While the importance and benefits of these programs have been proven, there is scant information in the literature and best-practice guidelines on the planning and development of these programs. This qualitative study focuses on the research, planning, and building development for new IDCs in metropolitan areas. It is based on a case example of the process of establishing an IDC in the City of Austin, which was an element of the Age-Friendly Austin Plan. It examines the applicable literature and the extensive involvement of experts in architecture, community planning, and public health policy as well as data collected from community engagement workshops to facilitate the IDC’s creation and operation. This study offers a developmental strategy method that can be adopted and utilized by other cities, developers, and designers who are interested in building IDCs.

## 1. Introduction 

The World Health Organization initiative of Age-Friendly Communities promotes municipal government efforts in developing social capital that encourages physical and social environments that meet older adults’ changing needs for assistance [1]. Programs that enhance the lives of older adults will have increasing importance as the U.S. population ages, and adult day care enhances social inclusion for older citizens while providing respite for informal caregivers [2]. Such caregivers are often also parents caring for the needs of their own children as well [3,4]. Given social change in which a middle-class lifestyle requires two incomes [5] and single parents must work to survive [6], no one is home during the day to care for dependent relatives. At the same time, municipal resources with which to provide care are scarce [7].

Intergenerational Day Centers (IDCs) are a newly emerging paradigm that combine day care services for older adults and children in one location. An IDC is a place where older adults and children spend their days while receiving ongoing services and programming at the same site and interact through planned and informal activities [8]. Combining services and spaces in this manner—potentially serving adults, youth, and young children—can have significant efficiencies [7]. Sharing spaces such as kitchen and dining areas and resources such as delivery and janitorial services saves money [9], lowering costs to families [10]. At a time when municipal governments face serious fiscal challenges potential savings could be significant. An intergenerational shared site promotes cost efficiency in terms of staff recruitment and training [11] and access to a wider range of fundraising opportunities than single-generation day care center can access [10]. IDCs address major community challenges, including (1) limited accessible and affordable healthcare for older adults [12], (2) affordability issues in both adult care [13] and childcare [14], and (3) institutional age-segregation due to systems being designed to serve one generation and exclude the other [15] that can lead to ageism and negatively impact older adults [16] and children [17]. In light of these advantages, cities are increasingly recognizing opportunities for the use of IDCs [18].

Interactions among generations through IDCs can be mutually beneficial. IDCs foster learning opportunities for both generations and increase social engagement among older adults and children who need not be family [8]. Intergenerational programs produce advancement in sensory stimulation; enhancement of self-esteem; increased positive socialization, and intellectual development for both older adults and children [9,19]. Intergenerational programs provide older adults with opportunities to use their life experience and expertise to develop and share activities such as cooking, science, and storytelling [20], to be childcare provider or partners in programs such as intergenerational theater [21]. Children involved in intergenerational programs are more prosocial and the nurturing presence of older adults helps bring a familial aspect to the preschool setting [22]. 

Although there is a need for intergenerational programs and the benefits of these programs have been observed [23,24,25], availability of IDCs is limited in Texas [26]. Additionally, based on the database of all the intergenerational day centers in the United States provided by Generations United, a national intergenerational membership organization representing over 500 agencies and individuals, there are only two IDCs, one in Alabama and one in Florida, among the six most southern states (Alabama, Arizona, Florida, Louisiana, New Mexico, Mississippi, Texas) of the United States. Furthermore, there is very limited information available in the literature or in other venues on how to plan, develop, and start a center for intergenerational interactions. In this study, we introduce the process of developing and intergenerational day center and offer a case study of research, planning, and building development of an IDC in a metropolitan area. The City of Austin has an increasingly older population, most of whom would prefer to age in place. Previous research highlights a growing need for community-based services, including wellness care and supportive services conveniently located all in one place for low-income families in Austin [27]. The IDC described here addresses this gap [28] and has significant implications for efforts in other communities.

## 2. Materials and Method

### 2.1. Study Procedure 

This study took place between 2018–2020, in three phases consisting of (1) an analytical literature review, (2) community engagement workshops, and (3) a feasibility study. 

### 2.2. Data Collection 

The research and data collection for the IDC center in Austin started in 2018 with a scoping literature review of reports of possible models of intergenerational day centers and the benefits for children, older adults, and the community. The study continued in 2019 in response to a City Council resolution that directed Austin Public Health (APH) to collaborate with the LBJ School of Public Affairs on a study of an IDC pilot located on city-owned property. IRB approval was received from The University of Texas for a study to gauge interest in creating an adult day care facility with integrated medical wellness clinic, comprehensive service coordination, and child daycare all in one place. In 2020, we conducted a feasibility study with the City of Austin that identified appropriate and specific IDC programs and services conforming to all city and state rules and regulations at the IDC.

#### 2.2.1. Analytical Literature Review 

Several databases were consulted, including Design and Applied Art Index, Taylor and Francis, and EBSCO. The research team members individually searched for and found peer reviewed articles based on the following keywords: intergenerational programs, design for all ages, intergenerational shared sites, intergenerational interactions, intergenerational space, and intergenerational architecture. The search resulted in a total of 15,149 articles, of which 100 were read. The process of elimination was through the title and abstract relevance in responding to the research question of this study. The 100 articles highlighted information about a study of intergenerational programs and strategies on the type of spaces that serve both older adults and children during intergenerational interactions. 

#### 2.2.2. Community Engagement Workshops

We then conducted an in-depth investigation (Table 1) on the needs of the community, seeking support from community members, policymakers, public sector organizations, collaborating with a public university, and inviting an intergenerational design specialist to join the team at early stages of the project. To achieve this, during Spring 2019, APH and the LBJ School gathered feedback through 79 community engagement sessions (CES) with Austin’s low-income older residents (n = 68), aging and respite service providers (n = 3), and informal caregivers (n = 9) to identify which services should be made available at the center. These workshops were conducted at three different locations of Mexican American Cultural Center, the Rebekah Baines Johnson(RBJ) Center for Living Center, Lakeside Senior Apartments in Central Austin. Five of the caregivers participated on line. The survey questions were available in English, Spanish, and Mandarin. Five of the caregivers participated online. In addition, 98 adults responded to five questions in a live poll at the Livability and Longevity Conference at UT Austin. Participants were given the opportunity to record their answers by using an i-clicker device, as an electronic means of counting responses. Participants who did not feel comfortable with the technology were provided a printed version of the survey. 

#### 2.2.3. Feasibility Study 

In 2020, a feasibility study was conducted to address the questions posed by APH: (1) What services could be provided in a pilot space that is 5000–10,000 square feet? And (2) What level of service is feasible based on the potential space and the city jurisdiction? 

To investigate the first question, the research team conducted personal interviews with older adults, children and caregivers using snowball sampling. Questions included the types and levels of intergenerational activities older adults and children most valued and enjoyed as well as what they wanted and needed from their intergenerational programs. The first author’s Team conducted 31 semi-structured interviews with 16 older adults and 15 children. To address the second question and determine feasible level of service for the planned space and city jurisdiction, the intergenerational design specialist used the results from literature review and the interviews to create schematic design documents including a floorplan and renderings that can be shared with and presented to the city officials and other stakeholders. 

### 2.3. Data Analysis

The analysis of the data collected for this study was conducted by two research teams working with the authors. The first author worked with two graduate assistants on the analytical literature review and the interview responses from older adults and children to determine spatial requirements for the IDC. The second author and a group of graduate students analyzed the findings from community engagement workshops. The final findings were reviewed and discussed by both authors to achieve agreements on all results. 

#### 2.3.1. Analytical Literature Review

Twenty-five of the 100 articles included detailed descriptions of spaces that best serve intergenerational programs. Reading and analysis of these articles resulted in 44 spaces within intergenerational programs that satisfy the needs of older adults and children during intergenerational activities. Examples of these spaces include multipurpose rooms, kitchens, courtyards, and classrooms. The research team individually read and analyzed each article and categorized them based on the type and level of intergenerational programs and interactions. The first category includes spaces built specifically for older adults such as adult day centers that later included intergenerational programs. These buildings are designed only for older adults and don’t generally consider the needs of children. The second category of intergenerational shared sites includes a child development center attached to an adult care center. The second category of intergenerational shared sites includes a child development center attached to an adult care center. These spaces are designed and built to accommodate the care and development of older adults and children while providing opportunities for different types of interactions simultaneously [29]. The third category includes community centers that are designed for people of all ages and promote temporary and informal interactions [30]. The spaces of this category are designed for public use, often with flexible and vibrant modular elements to create an energetic atmosphere for older adults and children to get together [30]. Places for intergenerational interactions within this category include multifunctional public spaces that benefit the community such as libraries and reading rooms, classrooms, indoor and outdoor gardens, parks, and outdoor playgrounds [31]. 

#### 2.3.2. Community Engagement Workshops

The research team used thematic content analysis to determine the presence of certain words, themes, or concepts within the qualitative data collected from the community engagement workshops. This helped the team to focus on the characteristics of language as communication with attention to the content or contextual meaning of the text and analyze the presence, meanings, and relationships of such certain words, themes, or concepts [32,33,34]. Responses were recorded on an Excel sheet and restored in Google doc. Analyses consisted of a collective reading of the data and a focused discussion in order to identify common themes and issues in which APH had inquired. Spanish and Mandarin interviews were translated into English by a subset of Team members who were fluent in the language. Both authors then reviewed the themes that resulted from the data until they reached 100% agreement.

#### 2.3.3. Feasibility Study

The research team transcribed the interviews and then conducted line by line coding by examining the data for thoughts, ideas, and issues mentioned by the interviewees [35]. These codes reflected the type of intergenerational activities older adults and children enjoyed which included being outdoors, cooking, and art. Focused coding as the next step provided clarity in choosing codes that best respond to the questions asked by APH about feasible services that can be provided in this IDC. 

The next phase of analysis was comparative theoretical coding among the findings from the literature analysis and feasibility study interviews. The research team wrote analytical memos to record the relationship between the datasets and the team’s interpretation of it. Through this step, we were able to check for definitions and specificity of the findings.

## 3. Results

This study focused on the process of developing and intergenerational day center in a municipal city with the goal of creating a sharing the process with other cities and organization who are also interested in starting an IDC. The steps taken for collecting information resulted in emergence of five major concentration of intergenerational programs, community needs assessment, spatial requirements, state regulatory requirements, and municipal government implementation that can be replicated as steps toward developing a new IDC center. Table 2 presents these concentrations along with specific findings from different data collection sessions. We provided these results to the city and other stakeholders in the form of several comprehensive reports and presentations [36]. 

### 3.1. Intergenerational Programs

The data collected from the interviews indicated that while individuals favored various intergenerational programs, most of these activities were focused under three main categories of art making, food-related activities, and shared outdoor activities. Participants described writing scripts, performing personal stories, playing musical instruments, dancing, creating hand puppets, sewing a quilt, and learning computer programs (e.g., how to have a video chat or send emails). 

### 3.2. Community Needs Assessment 

**Figure 1:**
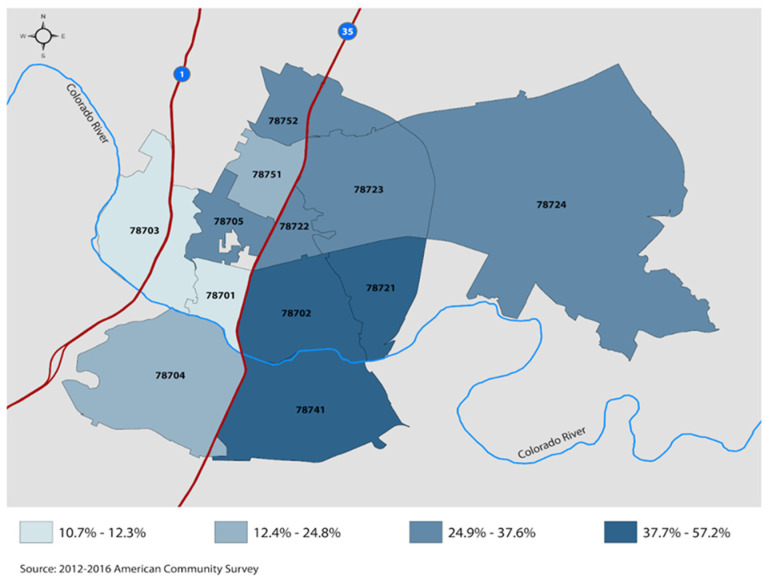
Place-Based Disparities in Aging of the City of Austin.

According to Central Health’s Planning Regions Overview, in the last decade Austin’s population 65 and over grew 44.2 percent [37]. (Central Health, 2015). This and the other community surveys we reviewed confirmed the need for an IDC near the RBJ Center. It also revealed that healthcare services are not readily available to low-income older adults. That need was emphasized by the 2013 Mayor’s Taskforce on Aging, which indicated that 40 percent of older adults in Central Texas worry they will not be able to pay for their healthcare and supportive care programs [38]. Figure 1 presents zip codes with high concentrations of older adults with incomes below 200% of poverty. The figure excludes richer zip codes. The data clearly illustrate the ecological concentration of need among older and vulnerable citizens. In addition to older individuals, families with children in these neighborhoods would also benefit from IDCs.

The results from the live poll revealed that among all respondents, close to 75 percent say they favor priority funding for older adults’ health, wellness, and social services all conveniently co-located in one place. Thirty-two percent agreed that Austin has a gap in wraparound services, such as affordable transportation, and 45 percent indicated that residents 65 and older should be entitled to public transportation based on a sliding income scale [39]. Our public input through community engagement workshops presented concerns about care and its costs for young children [40]. Over a third of children in the Austin Metropolitan Statistical area under age 6 live in low-income households and 90 percent are children of color. The vast majority, 80 percent, of single parents who earn less than 200% of the poverty line were employed either full or part time and 45% did not have access to affordable and high-quality childcare while 34% were unable to access early learning services for their children.

### 3.3. Spatial Requirements 

The findings of this study offered a better understanding of the preferences and spatial requirements for monogenerational and intergenerational activities. In order to develop an ideal architectural design solution for an IDC, we utilized multiple data sources gathered through engagement with end users and concluded that an ideal architectural design solution for IDCs supports intergenerational interactions that happen on a continuum from planned to spontaneous while offering individual choice and control over type and level of interaction. 

Intergenerational planned activities are generally developed and facilitated by staff members [20]. While they guarantee that intergenerational togetherness occurs [29], being compelled to participate together may meet resistance or cause resentment [8]. Spontaneous interactions have the advantage of involving fully willing participants and thus facility design should support their occurrence along with planned activities [22]. 

The 10,000 square-foot design we developed will support both planned and spontaneous interactions. Although a larger center would offer more opportunities for different types of intergenerational interactions, we deemed the 10,000 square feet model sufficient and aligned with the city’s budget. The design includes the following functional areas for group activities: a dividable multipurpose room for dining with adequate table setting space; an area for physical activities; a kitchen area for refrigerated food storage, the preparation of meals and/or training participants in activities of daily living; a quiet room (designed to hold at least one bed) to allow staff to isolate participants who become ill or disruptive, or who require rest, privacy, or observation; at least one toilet for every eight participants and equipped for persons with limited mobility; outside space that is safe, accessible to indoor areas, and accessible to those with a disability and that includes recreational space and outdoor workout equipment for adult exercise and learning areas for play and activities, and a garden area; and space for storage arts and crafts materials, personal clothing and belongings, wheelchairs, chairs, individual handiwork, and general supplies. Other space requirements are an individual room for counseling and interviewing participants and family members for tele-behavioral health screening and other matters and a reception area.

### 3.4. Municipal Government Implementation: Funding 

Our review of the existing literature resulted in different budgetary‘ needs and methods of fundraising [10]. Although, the federal government advocated for intergenerational programs, it is essential to approach individuals as well as government agencies. In regard to this project, under a proposed agreement the City of Austin will provide city-owned property and possible capital costs. The non-governmental organizations and private foundations will cover operational expenses.

## 4. Discussion

This study addresses the process of research, planning, and building development of an IDC center in a metropolitan area. The novelty of this study, aside from the fact that there is very limited existing information on this topic, lies on the presented data that emerged from different methods of literature review, community engagement workshops, and a feasibility study. We followed a design process that has been adopted by other local governments and incorporated the needs and experiences of the end users in the design solution. We addressed the challenges of setting up an IDC by focusing on a case example and all the steps the City of Austin has taken to bring sponsors together and create a plan that serves a wide range of needs. The study findings point to the importance of involving community members, stakeholders, and the design team in the development, site selection, architectural design decision making process, and programming plans as early as possible. This allows for complete transparency and advances the possibility of creating an IDC that responds to the end user’s needs. 

### 4.1. Interpretation of the Results 

According to the United States Census Bureau, while 33% of older adults have a disability, 41% of individuals 65 and older live in poverty and cannot afford their healthcare needs [41]. Simultaneously, the Austin’s family needs for affordable and high-quality childcare emphasizes the urgent necessity for early learning services for children in the Austin Metropolitan Statistical area. IDCs will provide not only co-located high-quality care for older adults and children, but also the required and requested healthcare, wellness, and social service. 

Interviewing older adults and children involved in intergenerational programs informed our decision on program development for both intergenerational activities and architectural design. One of the older adult interviewees said, “I am not an artist, but I enjoy painting…. [W]hen we make art with the kids, I get to teach them what I know.” Intergenerational food-related activities occurred daily at all three of the facilities that interviewees attended. Older adults talked about sharing family recipes with children and teaching children how to follow a recipe, safely handle food, and follow rules for table manners. Children enjoyed intergenerational gardening where they planted and grew vegetables with older adults and then used those vegetables to make a salad and share it over lunch. Other shared outdoor activities included bowling under a pavilion, and collecting leaves, and older adults described walking outside to visit children playing in the playground [42].

**Figure 2:**
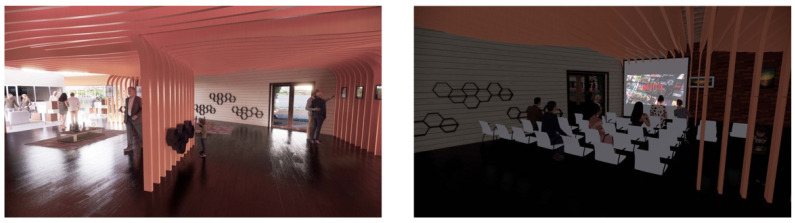
Central Multipurpose Space.

The architectural design of our IDC is structured to encourage social interaction between older adults and children while respecting their autonomy and desires and offering space for monogenerational activities. A large flexible space (Figure 2) connects the child development and the older adults’ sections for intergenerational activities. This space can also be utilized by the community on weekends, evening and when needed. It is flexible in the sense that it can be used for events, social gathering, a music and dancing platform, and an art gallery to showcase art created by older adults and children. 

**Figure 3:**
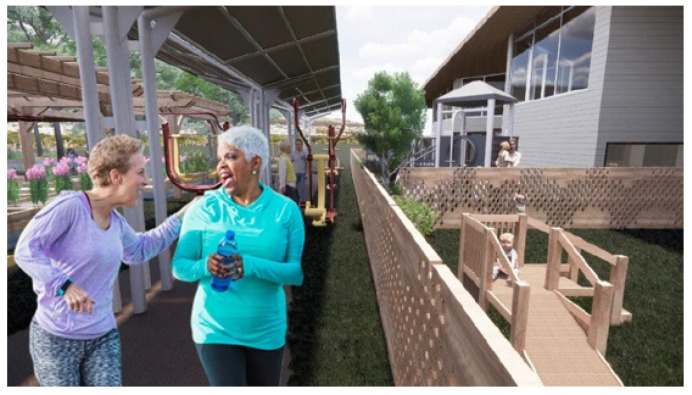
Outdoor fitness area for older adults is adjacent to children’s playground.

Outdoor space is designed to offer three different levels of interaction: (1) visual, (2) visual and auditory, and (3) being together in the same space. For example, visual interaction can be provided through observation windows outside the children’s classrooms to offer older adults the option to watch the children play. An outdoor fitness center for older adults that is adjacent to the children’s playground potentially offers visual and auditory interaction (Figure 3). 

**Figure 4:**
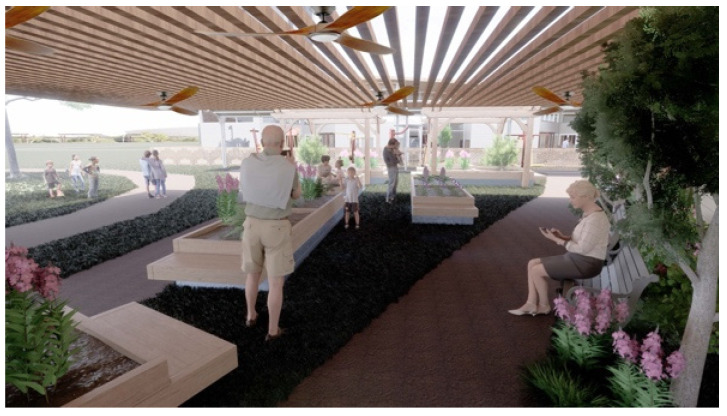
Outdoor Garden adjacent to outdoor fitness area.

Observing children’s energy in the playground can inspire older adults to spend more time outside and use the exercise equipment while watching and listening to children play [34]. Surrounding the outdoor fitness area with greenery (Figure 4) also creates a harmonious experience for older adults in the space and gives them options when they are in this area. 

Both the architectural design drawings and the data collected in this study were used by City Council who executed Resolution item #59 (20221208-59) on December 8, 2022. The resolution directs the City Manager to work with the IDC Advisory Group that consists of community volunteers and city staff on the project. The goal is to finalize a proposal for Council to fund an Intergenerational Resource and Activity Center.

### 4.2. Strength and Limitations

This study has many strengths. First, it is one of the few studies that has examined the political and practical challenges of implementing an intergenerational day center designed specifically for low-income seniors who have limited access to such care. Second, the research focuses on the potential role of municipalities and non-governmental actors in establishing such a facility close to affordable senior housing. Third, in addition to increasing access for low-income older adults, the intergenerational aspect of care both optimizes the use of space and potentially enhances social development for children and improves older adults’ cognitive and social abilities. Finally, our multi-method approach— personal interviews, focus groups, household surveys, and polling involving input from low-income older adults (co-production) provides multiple sources of information that helps us asses the utility of new ways of providing community-based long-term services and supports for caregivers, care recipients, providers, and the city of Austin. Our study presents a strategy method that is the first model of its type and can be adopted and utilized as the foundation for other cities, developers, and designers who are interested in establishing IDCs. A clear limitation of this study is that it is based on one municipality and since the building has not yet been completed political difficulties may intervene. Also, to our knowledge, there are currently no other published model[s] to compare with this method.

### 4.3. Future Research Direction

Our model for developing an IDC is focused on a vision that provides health and wellness support. However, the range of possible intergenerational programs, settings, and participants are as diverse as the communities and their needs, as well as the implementation of the program and how the organizations work. Future research should focus on the developmental process for other types of intergenerational centers. Additionally, future research should evaluate how such programs can best be adapted to the unique needs of other municipalities that confront the need for infant day care and the need to provide long-term care services that supports to vulnerable older individuals.

## 5. Conclusions

Our findings support the value of IDCs in metropolitan areas. Complementing our analytical review of the existing literature, the data we collected from interviews, community engagement workshops, governments and non-government documents and collaborations demonstrate that IDCs have individual, organizational, and societal benefits. IDCs afford sharing spaces and resources while offering easy transportation that makes it easier for administrators, program facilitators and caregivers to bring older adults and children together, which leads to more frequent and sustained interaction through planned and spontaneous interaction between older adults and children. Thus potentially, lowering the cost of care for families who need day care services for older adults and/or children. 

The experience presented in this study indicates that adoption of IDCs relies on motivated constituencies, including an effective advocacy body, political will among leaders at various levels of government, and adherence to local cultural norms. All stakeholders should be included in the process of creating an IDC and be involved in all major decision making so that IDCs serve their goal of supporting the needs of all the generations they serve. The design model created based on our findings supports the value of a developmental strategy method and can be adopted and utilized by other cities, developers, and designers who are interested in building IDCs.

## Figures and Tables

**Table 1:** IDC Pilot Feasibility Study Data.

Local Parties Involved	Sources of Information
Community Members	Personal &Telephone Interviews & Surveys
Policy Makers	Academics
Public Sector Organizations	Government & Local Documents
Austin Public Health	Administrative Memoranda
Intergenerational Design Specialist	Consulting with Experts
The University of Texas at Austin	Site Assessments

**Table 2:** IDC Feasibility Results.

Communication & Data Collection Method	Concentration	Findings
Personal Interviews	Intergenerational programs	Art making Food-related activities Shared outdoor activities
Community surveys (e.g., live poll)	Community needs assessment	Required healthcare services Wellness services Social services
Analytic Literature Review	Spatial requirements	Monogenerational activities Intergenerational activities Administrative and staff
Inspection of Government Documents	Texas state regulatory requirements	Social activities Nutrition & food services Nursing services & physical rehabilitation Transportation to & from the center
Austin Public Health & Non-governmental providers	Municipal Government Implementation	Program development & community engagement cost Architectural design & construction cost Operational & maintenance cost Endowment/reserve fund

## Data Availability

The data are available from the second author.

## References

[1] Phillipson C., Richard J., Settersten A., Angel J.L. (2011). Developing Age-Friendly Communities: New Approaches to Growing Old in Urban Environments. Handbook of Sociology of Aging.

[2] Scharlach A., Lehning A. (2013). Ageing-friendly communities and social inclusion in the United States of America. Ageing Soc..

[3] Salari S. (2002). Intergenerational Partnerships in Adult Day Centers: Importance of Age-Appropriate Environments and Behaviors. Gerontologist.

[4] Buffel T., Phillipson C. (2018). A Manifesto for the Age-Friendly Movement: Developing a New Urban Agenda. J. Aging Soc. Policy.

[5] Bookings. https://www.brookings.edu/essay/the-history-of-womens-work-and-wages-and-how-it-has-created-success-for-us-all/.

[6] United States Census Bureau. https://www.bls.gov/news.release/pdf/famee.pdf.

[7] Urban. https://www.urban.org/sites/default/files/publication/101441/incorporating_two-generation_approaches_in_community_change_2_5.pdf.

[8] Norouzi N., Swenson A., Harvey S. (2022). Designing for Success: Integrating Theories of Human Development into Architectural Design for Intergenerational Programming. J. Intergenerational Relatsh..

[9] Jarrott E.S., Bruno K. (2007). Shared site intergenerational program: A case study. J. Appl. Gerontol..

[10] (2021). Generations United. http://www.sharingourspace.org/funding/.

[11] (2019). Generations United. https://www.gu.org/app/uploads/2019/11/Intergenerational-Shared-Site-Funding-Paper.pdf.

[12] Okoro C., Hollis N.D., Cyrus A.C., Griffin-Blake S. (2018). Prevalence of Disabilities and Health Care Access by Disability Status and Type Among Adults—United States, 2016. Morb. Mortal. Wkly. Rep..

[13] Beach S.R., Schulz R., Friedman E.M., Rodakowski J., Martsolf R.G., James A.E. (2020). Adverse Consequences of Unmet Needs for Care in High-Need/High-Cost Older Adults. J. Gerontol. Ser. B Psychol. Sci. Soc. Sci..

[14] (2014). Child Care Aware of America. https://files.eric.ed.gov/fulltext/ED559901.pdf.

[15] Hagestad G.O., Uhlenberg P. (2006). Should We Be Concerned About Age Segregation? Some Theoretical and Empirical Explorations. Res. Aging.

[16] Fried L.P., Carlson M.C., McGill S., Seeman T., Xue Q.-L., Frick K., Tan E., Tanner E.K., Barron J., Frangakis C. (2013). Experience Corps: A dual trial to promote the health of older adults and children’s academic success. Contemp. Clin. Trials.

[17] Levy B.R. (2009). Stereotype embodiment: A psychosocial approach to aging. Curr. Dir. Psychol. Sci. A J. Am. Psychol. Soc..

[18] (2021). AARP.com. https://aarp-states.brightspotcdn.com/2b/72/be52b3de4595a1fb331ffb711439/age-friendly-austin-progress-report-2021-final.pdf.

[19] Heydon R. (2013). Learning at the Ends of life: Children, Elders, and Literacies in Intergenerational Curricula.

[20] Norouzi N., Chen J., Jarrott S. (2015). Intergenerational explorations: Where everyone has a purpose. J. Intergenerational Relatsh..

[21] Norouzi N., Lyon-Hill S. “All the World’s a Stage”—Bridging the Generational Gap through Theatre. Proceedings of the Gerontological Society of America Conference.

[22] Norouzi N. (2016). Intergenerational Facilities: Designing Intergenerational Space through a Human Development Lens. Doctoral Dissertation.

[23] Giraudeau C., Bailly N. (2019). Intergenerational programs: What can school-age children and older people expect from them? A systematic review. Eur. J. Ageing.

[24] Lee K., Jarrott S.E., Juckett L.A. (2020). Documented Outcomes for Older Adults in Intergenerational Programming: A Scoping Review. J. Intergenerational Relatsh..

[25] Kaplan M., Sanchez M., Hoffman J. (2017). Intergenerational Pathways to a Sustainable Society.

[26] Generations United (n.d.) Intergenerational Program Database. https://www.gu.org/ig-program-database/?search=Intergenerational%20day%20center&state=TX&topic=intergenerational-programs-space.

[27] (2020). LBJ School of Public Affairs, The University of Texas at Austin. https://repositories.lib.utexas.edu/bitstream/handle/2152/65188/Final%20Report%20Intergenerational%20Day%20Center%20Pilot%20July%2016%202020.pdf?sequence=25&isAllowed=y.

[28] (2018). LBJ School of Public Affairs, The University of Texas at Austin. https://repositories.lib.utexas.edu/bitstream/handle/2152/65188/PRP%20188%20Angel.pdf?sequence=20&isAllowed=y.

[29] Norouzi N., Jarrott S., Chaudhury H. (2019). Designing intergenerational space through a human-development lens. J. Archit. Plan. Res..

[30] Ter L.V., Isa M.H.M. (2020). Architecture Spaces to Promote Intergenerational-Friendly Environment. MAJ-Malays. Archit. J..

[31] Kaplan Thang L., Sánchez M., Hoffman J. (2020). Intergenerational Contact Zones: Place-Based Strategies for Promoting Social Inclusion and Belonging.

[32] Weber R. (1990). Basic Content Analysis.

[33] Smith C. (1992). Motivation and Personality: Handbook of Thematic Content Analysis.

[34] Hsieh H., Shannon S.E. (2005). Three Approaches to Qualitative Content Analysis. Qual. Health Res..

[35] Charmaz K., Holstein J.A., Gubrium J.F. (2008). Constructionism and the grounded theory method. Handbook of Constructionist Research.

[36] (2018). The University of Texas at Austin. https://repositories.lib.utexas.edu/handle/2152/65188.

[37] Central Health (2015). Central Health Planning Regions Overview, 2014–19: An analysis of Age, Poverty and Race/Ethnicity Trends in Travis County. https://www.centralhealth.net/wp-content/uploads/2015/10/Demographics-FINAL-web.pdf.

[38] Mayor’s Task Force on Aging Report and Recommendations 2013. https://www.austintexas.gov/sites/default/files/files/Council/Mayor/Mayor_s_Task_Force_on_Aging_Full_Report.pdf.

[39] (2019). LBJ School of Public Affairs, The University of Texas at Austin. https://repositories.lib.utexas.edu/bitstream/handle/2152/65188/MMAC%20Resolution%2020181018-041%20Austin%20Public%20Health.pdf?sequence=36&isAllowed=y.

[40] (2020). Success by 6. https://www.austintexas.gov/edims/document.cfm?id=336077.

[41] (2015). United States Census Bureau. https://www.census.gov/newsroom/press-kits/2015/acs_oneyear.html.

[42] Norouzi N., Chen J.C., Jarrott S., Sattari A. (2022). Designing Intergenerational Spaces: What to Learn from Children. HERD Health Environ. Res. Des. J..

